# Qualitative Theory of Differential Equations, Difference Equations, and Dynamic Equations on Time Scales

**DOI:** 10.1155/2016/9094613

**Published:** 2016-10-26

**Authors:** Tongxing Li, Martin Bohner, Tuncay Candan, Yuriy V. Rogovchenko, Qi-Ru Wang

**Affiliations:** ^1^School of Informatics, Linyi University, Linyi, Shandong 276005, China; ^2^Department of Mathematics and Statistics, Missouri S&T, Rolla, MO 65409-0020, USA; ^3^Department of Mathematics, Faculty of Arts and Sciences, Niğde University, 51200 Niğde, Turkey; ^4^Department of Mathematical Sciences, University of Agder, Postboks 422, 4604 Kristiansand, Norway; ^5^School of Mathematics, Sun Yat-sen University, Guangzhou 510275, China

We are pleased to present this special issue. This volume reflects an increasing interest in the analysis of qualitative behavior of solutions to differential equations, difference equations, and dynamic equations on time scales. Numerous applications arising in the engineering and natural sciences call for the development of new efficient methods and for the modification and refinement of known techniques that should be adjusted for the analysis of new classes of problems. The twofold goal of this special issue is to reflect both the state-of-the-art theoretical research and important recent advances in the solution of applied problems.

The call for papers prepared by the guest editors encouraged submission of contributions on a wide spectrum of topics including asymptotic behavior of solutions, existence of periodic and almost periodic solutions, solvability of boundary value problems, stability properties of solutions, and applications to real world phenomena. In response to this call, twenty manuscripts addressing important problems in related areas were submitted to the editorial office and went through a thorough peer refereeing process. Six research articles reflecting modern trends and advances in differential equations have been selected for this special issue.

The paper by K. L. Cheung and S. Wong is concerned with the analysis of the blowup phenomenon in the initial-boundary value problem for *N*-dimensional Euler equations with spherical symmetry. Y. Li et al. introduce the notion of weak *𝒟*-pullback exponential attractor for a class of nonautonomous dynamical systems and suggest a general method for proving the existence of a weak *𝒟*-pullback exponential attractor. F. Sánchez-Garduño and J. Pérez-Velázquez study the existence of traveling wave solutions for a class of one-dimensional nonlinear degenerate reaction-diffusion-advection equations. The paper by H. S. Mahato analyzes transmission properties of a metallic layer with narrow slits. Using the implicit function theorem and implicit derivatives, T. Lindström and Y. Cheng prove that a well-known Rosenzweig-MacArthur graphical criterion for local stability holds also under chemostat conditions. A. Shatyrko and D. Khusainov establish sufficient conditions for absolute stability and interval absolute stability of direct control systems described by first-order delay differential equations and neutral delay differential equations.

The editors hope that this collection of papers will attract interest of researchers working in related areas and will stimulate further progress in the qualitative theory of differential equations, difference equations, and dynamic equations on time scales.

